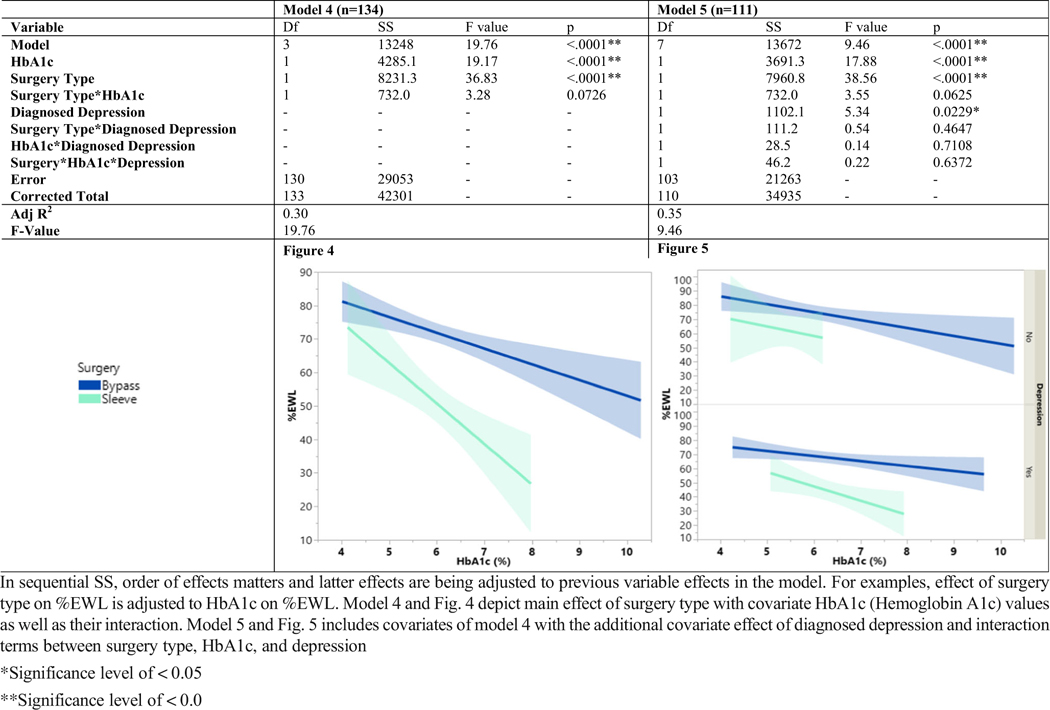# Bariatric Surgery Outcomes in Appalachia Influenced by Surgery Type, Diabetes, and Depression

**DOI:** 10.1007/s11695-018-03650-1

**Published:** 2019-04

**Authors:** Makenzie L. Barr, Lawrence E. Tabone, Stephanie J. Cox, Cassie Brode, Nova Szoka, I. Mark Olfert, Laura Davisson, Melissa D. Olfert

**Affiliations:** 1 Department of Human Nutrition and Food, Division of Animal and Nutritional Sciences, Davis College of Agriculture, Natural Resources, and Design, West Virginia University, G25 Agriculture Sciences Building, 333 Evansdale Dr, Morgantown, WV 26506, USA; 2 Department of Surgery, West Virginia University School of Medicine, Morgantown, WV, USA; 3 Department of Medicine, West Virginia University School of Medicine, Morgantown, WV, USA; 4 Department of Exercise Physiology, West Virginia University School of Medicine, Morgantown, WV, USA

**Keywords:** Obesity, Bariatric surgery, Appalachia, Outcomes

## Abstract

**Background:**

Most effective treatment for morbid obesity and its comorbidities is bariatric surgery. However, research is limited on weight loss and associated outcomes among patients in Appalachia. The objective of this study was to examine demographic and comorbidity influence on surgical outcomes of this population including age, sex, race, state of residence, education, marital status, body mass index (BMI kg/m^2^), excess body weight (EBW), percent excess weight loss (%EWL), blood pressure, diagnosed depression, diagnosed type 2 diabetes (T2D), Beck Depression Inventory-II (BDI-II), and laboratory values (i.e., hemoglobin A1c).

**Methods:**

A retrospective electronic medical record (EMR) data extraction was performed on *N* = 582 patients receiving bariatric surgery (laparoscopic Roux-en-Y gastric bypass [RYGB] and laparoscopic sleeve gastrectomy [SG]) between 10/2013 and 2/2017.

**Results:**

Patient population was 92.5% Caucasian, 79.3% female, 62.8% married, 45 ± 11.1 years, 75.8% received RYGB, and 24.2% received SG. Average %EWL from baseline to 1-year follow-up was 68.5 ± 18.4% (*n* = 224). In final descriptive models, surgery type, diagnosed T2D, HbA1c, and depressive symptoms were significant covariates associated with lower %EWL.

**Conclusions:**

Findings suggest patients completing surgery within an Appalachian region have successful surgical outcomes at 1-year post-surgery, as indicated by significant reductions of > 50% EWL, regardless of other covariates. Results suggest that bariatric programs should consider paying special consideration to patients with T2D or depressive symptoms to improve outcomes. Results have potential to inform future prospective studies and aid in guiding specific interventions tailored to address needs of this unique population.

## Introduction

The Appalachian region has dramatic health disparities [1, 2] reflected in the prevalence of type 2 diabetes (T2D), heart disease, obesity, and mental illness in conjunction with economic and infrastructure disparities [2–4]. The prevalence of obesity within the Appalachian region is among the highest in the world. Traditionally, obesity treatment has focused on behavioral, dietary, and lifestyle interventions that are employed on a community-based level.

For individuals with class III obesity (BMI ≥ 40 kg/m^2^), which is over 6% of the US population, behavioral interventions alone are often non-therapeutic, resulting in marginal sustained weight loss and poor comorbidity resolution [5–8].

Metabolic and bariatric surgery has been proven to be the most effective treatment for class II and III obesity and yet remains highly underutilized [9–13]. Primary bariatric procedures performed in the USA include laparoscopic Roux-en-Y gastric bypass (RYGB) and laparoscopic sleeve gastrectomy (SG) [9, 11, 14–16]. Between 2014 and 2017, total amount of surgeries in the USA increased from 193,000 to 228,000 with RYGB currently making up 17.8% and SG making up 59.4% [15, 16]. Of note, across the same years, RYGB surgeries have declined (− 9.0%) while SG procedures increased (+ 7.7%) [15, 16]. Bariatric surgery results in a number of positive outcomes such as significant reductions in excess body weight and declines or remission of comorbidities (T2D, improved quality of life, hypertension, gastrointestinal reflux disease, depression, and others). As such, bariatric surgery represents the most effective treatment for individuals with morbid obesity [17–20].

However, in Appalachia, a region with high obesity prevalence and related health disparities, there is a gap in the research regarding bariatric surgery patient populations and their surgical and related outcomes. An article by Bergmann et al. examined how the rural status of bariatric surgery patients impacted their access to and outcomes of surgery [21]. This study found that in patients having surgery, rural status (based on Rural-Urban Commuting Areas) did not have a relationship with surgical weight outcomes or compliance with follow-up appointments at 1 year post-operatively. However, insurance was a confounding factor in the study and often barred rural individuals from obtaining surgery [21]. Additionally, Mock et al. [22] examined limited food budgets among bariatric patients and found a significant reduction in weight loss outcomes when on a limited budget at 3-month post-bariatric surgery. However, that significance was not found at 12-month post-bariatric surgery [22]. These studies highlight the importance of how variables such as baseline patient health and demographics may influence outcomes. However, understanding the impact of health disparities on the outcomes of bariatric/metabolic surgery is also vital to mitigating the numerous barriers faced by patients.

Thus, the objective of the current study is to expand the knowledge base of bariatric surgery patients located in a health disparate region of Appalachia. Specifically, the goal of this study was to examine demographic, surgical/medical/laboratory, weight outcomes from pre-surgery to 1-year post-surgery and the influence baseline health measures had on surgical outcomes. To our knowledge, this is the first study to comprehensively describe a large Appalachian bariatric surgery patient population and their surgical outcomes.

## Materials and Methods

A retrospective chart review was performed of a high volume bariatric surgical program in a tertiary university hospital that provides care to the Appalachian population. Approval to conduct research was obtained via [Institution removed for blinding purposes] Institutional Review Board (IRB Protocol #1611355277). A patient query was completed on patients, 18 years and older, who had completed all required clearances (i.e., cardiovascular, pulmonology, psychology) and received RYGB or SG surgery between October 1st, 2013 and December 31st, 2017. Formal consent was not required. Patient surgeries at this clinic are > 99% funded through insurance. Retrieval of information was found in forms of both electronically entered data as well as scanned and uploaded PDF files. Uploaded PDF files included self-completed forms from patient’s initial clinic visits (i.e., nutrition history, health, and family history). All data were entered into a HIPAA compliant RedCap survey and downloaded onto a secure, password protected, encrypted hard drive for further data analyses. A second data pass was completed on 2% of charts to ensure data reliability of 85%.

### Study Measures

Patient electronic medical record (EMR) data were captured at patients’ baseline clinic visit(s) for bariatric surgery with a bariatric surgeon, dietician, nurse practitioner, physician’s assistant, and psychologist. Baseline demographics, anthropometrics, lab results (i.e., hemoglobin A1c), health history, family history, nutrition habits, and psychological testing scores (i.e., depression symptoms) were recorded at the time of these visits. Changes in anthropometrics obtained through 1-year, post-surgery, follow-up visits were logged in patients’ EMR. The main outcome measure was percent excess weight loss (%EWL) from baseline to 1-year follow-up with an ideal body weight representing a BMI of 25 kg/m^2^. As used in other studies, a %EWL of 50% or more achieved within 12 months of surgery was considered a therapeutic success. Predictor variables used include surgery type, age, gender, ethnicity, education, marital status, percent follow-up attendance (number of follow-ups attended/determined by amount of follow-ups possible multiplied by 100), diagnosed hypertension, diagnosed depression (defined as any ICD-10 depression diagnosis listed in patients’ charts as assigned by their providers), and cooking responsibilities. HemoglobinA1c (HbA1c) values, blood pressure values, and Beck Depression Inventory II (BDI-II) scores were collected in addition to physician-recorded diagnoses. These measures were also used as predictor variables in separate models to examine ICD-10 diagnoses compared to other measurement forms to assess blood glucose control, blood pressure, and self-reported de-pressive measures.

### Statistical Analyses

All analyses were performed using SAS (SAS®, Version 9.3) [23] and JMP (JMP®, Version Pro 13) [24]. Data were examined for variable-specific outliers greater than 3 standard deviations above the mean, which were removed prior to analyses (*n* = 10 outliers). Differences were tested between baseline measures of surgery groups (RYGB vs. SG). An independent *t* test was used for assessing association between %EWL and variables with two groups (surgery type, gender, ethnicity, education level, state, marital status, diagnosed T2D, diagnosed hypertension, diagnosed depression). ANOVA was used for testing the hypothesis of equality among more than two groups of categorical variables (education and marital status), and Spearman’s Rho was used for examining correlations of %EWL with continuous variables (age, % attended follow-up, systolic blood pressure, diastolic blood pressure, HbA1c, and BDI-II score). Fisher’s exact test was used for cell sizes < 5. Significant correlations of *p* < 0.05 were included in the next step of building ANOVA and ANCOVA models to test relationships between %EWL and categorical and continuous predictor variables. ANOVA models tested ICD-10 diagnoses of diabetes, hypertension, and depression. ANCOVA models tested HbA1c lab values, blood pressure readings, and BDI-II scores as secondary measures. Model assumptions of homoscedasticity, normality, and lack of multicolinnearity were assessed. Cook’s D influence was set at 0.0227 (4/n). Data with an influence greater than Cook’s D were removed from analysis (*n* = 7). Effect size in models was assessed by change in adjusted *R*^2^ values to calculate variance of each variable when placed in the model. ANOVA models were computed using PROC MIXED procedure Type III Sum of Squares (SS) in SAS (partial), and ANCOVA models were computed using PROC GLM procedure Type I SS (sequential). In partial SS, the hypothesis to be tested are invariant to the ordering of effects in the model. In sequential SS, order of effects matters, and latter effects are being adjusted to previous variable effects in the model. For example, effect of surgery type on %EWL is adjusted to HbA1c on %EWL. Effect size in final models was assessed by change in adjusted *R*^2^ values to calculate variance of each variable when placed in the model. Stepwise modeling was used for ANOVA and ANCOVA tests to allow results of each variable to be shown through their individual extent of influence in relationship to %EWL. Further, utilizing both categorical indicators of diagnosed diabetes and depression as well as continuous indicators to remove caution of differences among outcomes when using categorical or continuous variables.

## Results

From the initial query, data was captured on a total of 582 patient charts. Sample size was based on time and data limitations. Thirty-five charts and corresponding data were removed due to type of surgery being a gastric band or revision of previous surgery leaving a sample of *n* = 547. The bariatric surgery patients were predominately 92.5% Caucasian, 79.3% female, 62.8% married, 45 ± 11.1 years old, and 75.8% receiving RYGB surgery. When stratifying the population by surgery type, similar demographic breakdowns were seen. No significant demographic differences were found between two surgery type groups (Table 1). RYGB patients had an average baseline weight of 136.5 ± 29.2 kg, BMI of 48.5 ± 8.1 kg/m^2^, and EBW of 66.7 ± 25.3 kg. SG patients had average baseline weight of 139.5 ± 26.5 kg, BMI of 49.2 ± 8.0 kg/m^2^, and EBW of 69.0 ± 23.8 kg. The EMR was examined for the following reported baseline comorbidities: diagnosed T2D (*n* = 174), diagnosed depression (*n* = 259), and diagnosed hypertension (*n* = 304). Among these comorbidities, no significant differences were found among groups at baseline (Table 1; all *p* > 0.05). Likewise, no significant differences between surgery groups were found among objective measures of HbA1c, blood pressure, and BDI scores (all *p* values > 0.05). Percent follow-up at 1-year appointment was 47% for bypass (*n* = 196) and 30% for sleeve patients (*n* = 40) (*p* < 0.001). Average %EWL among whole sample was 68.80 ± 18.92% with bypass patients achieving higher %EWL than their sleeve counterparts (*p* < 0.0001).

Bivariate analyses identified 6 of 15 dependent variables of interest that had significant associations (*p* < 0.05) with %EWL (Table 2). Surgery type, age, diagnosed T2D, diagnosed depression, diagnosed hypertension, and HbA1c values all found to have a significant association with %EWL (Table 2; all *p* < 0.05). As HbA1c and diagnosed T2D were both significantly related to %EWL, separate models were used to display their effect as they both describe abnormal glucose control. Variables were utilized in further model building to test the influence of each significant identified variable on predicting %EWL at 1-year post-bariatric surgery. In a preliminary full screening model, surgery type, diagnosed T2D, depression, and hypertension, and HbA1c value remained significant (Table 2). To further analyze variance of %EWL caused by remaining significant variables ANOVA and ANCOVA models were built (Tables 3 and 4; Figs. 1–5). Both HbA1c and Diagnosed T2D measured blood glucose control status and thus, separate models were designed for both

Model 1: %EWL = surgery type

Model 2: %EWL = surgery type + diagnosed T2D

Model 3: %EWL = surgery type + HbA1c

Model 4: %EWL = surgery type + diagnosed T2D + diagnosed depression

Model 5: %EWL = surgery type + HbA1c + diagnosed depression

Model 1 examines the main effect of surgery alone on %EWL (*F* (1,222) = 45.72, *p* < 0.0001). Figure 1 shows significantly higher %EWL in bypass patients (72.16 ± 17.44 %EWL) compared to sleeve (51.15 ± 16.62 %EWL) at 1-year follow-up (*p* < 0.001). Effect of diagnosed T2D, surgery, and interaction between T2D and surgery on %EWL is depicted in model 2. Type 3 fixed effects for both surgery (*F* (1,199) = 44.95, *p* < 0.0001) and EMR-diagnosed T2D were significant (*F* (1,199) = 15.49, *p* = 0.0001). Fig. 2 represents the main effect of surgery type although interaction between the two were not significant (*F* (1,199) = 0.38, *p* = 0.5368). Model 3 examines surgery type, EMR-diagnosed T2D, and EMR-diagnosed depression and their interactions. Type 3 fixed effects identify significance among surgery type (*F* (1,170) = 15.88, *p* < 0.0001), diagnosed T2D (*F* (1,170) = 5.59, *p* = 0.0192), as well as diagnosed depression (*F* (1,170) = 8.37, *p* = 0.0043). All interaction terms between each combination of surgery, T2D, and depression were found as non-significant (*p* > 0.05).

Among models 4 and 5 (Table 4), diagnosed T2D was replaced with objective measure of HbA1c blood glucose control. Model 4 (*F* (3,133) = 9.46, *p* < 0.0001), examined main effect of surgery on %EWL while controlling for HbA1c. Model 4 had an *R*-squared value of 0.31 and found both surgery type and HbA1c had significant relationship with %EWL (*p*’s < .0001); however, interaction term between surgery and HbA1c was not significant (*p* = 0.07). Model 5 (*F* (7, 110) = 9.46, *p* < 0.0001) with an *R*-squared value of 0.39, examined main effect of surgery type on %EWL while controlling for HbA1c and diagnosed depression. Variables of surgery type (*p* < 0.0001), HbA1c (*p* < 0.0001), and diagnosed depression (*p* = 0.0229) were all significant; however, all interaction combinations were not statistically significant (*p* > 0.05).

## Discussion

In this sample of Appalachian RYGB and SG patients, 1-year weight loss outcomes were comparable to and exceeded those in the current literature. Various studies and reviews identify bariatric surgery as aiding in the success of 40–71% EWL post-surgery [14]. Within each ANOVA and ANCOVA model in this study, SG patients typically had less %EWL than RYGB patients. Additionally, we found a trend that patients with a diagnosis of T2D or depression, according to ICD-10 codes, achieved lower %EWL. We were able to identify that each variable separately (surgery type, diagnosed T2D, elevated HbA1c, and diagnosed depression) impacts %EWL. Generally, those receiving SG, being diagnosed with T2D or depression, or having a higher HbA1c at baseline had lower %EWL at 1-year post-op. Our data is consistent with previous data. Specifically, in patients with T2D receiving SG, patients had a 47% EWL which is similar to our findings [25]. The well-known Swedish Obese Subject (SOS) study examined longitudinal weight among surgical patients [20, 26, 27]. However, a limitation of the SOS is that no SG procedures were performed. In a 2003–2015 registry reported by the International Federation for Surgery for Obesity and Metabolic Disorders, 49.4% received RYGB followed by 40.7% receiving SG [28]. Total body weight loss at 1-year follow-up in this registry population was 30% [28]. A similar study by Shah et al. examined retrospective data of bypass and sleeve patients. Outcomes of > 50% EWL were seen more frequently in those patients who had lower initial BMI, absence of T2D, and underwent RYGB surgery [29]. Our population data shows similar results as compared to these listed studies and national averages for percent excess body weight loss 1 year after surgery. With this study being the first to our knowledge to examine an Appalachian centered population, more work is warranted to determine whether these results can be replicated in other rural regions and settings.

Due to the retrospective nature of data retrieval, some data could not be captured through the patient EMR. For example, forms that were hand-written and scanned into the chart were, at times, illegible, which led to incomplete data. Further, our patient population was primarily Caucasian females who received RYGB. Of these patients, a significantly higher amount of bypass patients returned for 1-year follow-up as compared to SG patients. Due to this outcome, caution needs to be taken with results based on lack of follow-up in SG, generalizability to a larger study population attention should be taken. Had a larger percentage of SG returned for follow-up appointments, outcomes may have reflected differently. However, overall, this population demographic is largely representative and similar to that of the nation’s bariatric surgery demographic breakdown.

In summary, this study found that although patients may reside in a health disparate location such as Appalachia, bariatric and metabolic surgeries can still be successful for achieving significant weight loss after 1-year follow-up. Specifically, results indicate when utilizing SG for weight loss surgery in this geographical region, successful outcomes may be less frequent when patients have additional comorbidities. However, overall consideration needs to be taken when supporting individuals with obesity related comorbidities such as T2D and depression because those factors were associated with lower %EWL. Therefore, it is recommended that health practitioners/public health experts endorse metabolic surgery for populations who are morbidly obese, specifically in Appalachian regions, as well as support individuals with comorbidities with additional resources for success, particularly among SG. However, due to limited longitudinal data regarding this population, future research examining success of behavioral and dietary patterns as well as comorbidity resolution are warranted.

## Figures and Tables

**Table 1:** Descriptive statistics, by surgery type, of Appalachian bariatric surgery patients between 2013 and 2017 receiving surgery in West Virginia

Variable		*n*	Bypass	*n*	Sleeve	*p* value
Demographics (*n* = 547)						
Sex	Male	407	81 (20)	128	29 (22.7)	0.5012
	Female		326 (80)		99 (77.3)	
State	West Virginia	406	326 (80.3)	128	104 (81.3)	0.8121
	Other		80 (19.7)		24 (18.7)	
Race	Caucasian only	407	376 (92.4)	127	118 (92.9)	1.0000
	Other		31 (7.6)		9 (7.1)	
Education	High School or less	386	149 (38.6)	125	40 (32.0)	0.4079
	Some College or Associates		131 (33.9)		44 (35.2)	
	Bachelors		67 (17.4)		29 (23.2)	
	Post Grad, Masters, PhD, Law		39 (10.1)		12 (9.6)	
Marital	Single	377	65 (17.2)	117	22 (18.8)	0.8468
	Married		235 (62.3)		74 (63.2)	
	Divorced		53 (14.1)		16 (13.7)	
	Other		24 (6.4)		5 (4.3)	
Diagnosed baseline comorbidities		*n*	%	*n*	%	
	T2D	138	37.3	36	30.0	0.1414
	Hypertension	227	64.9	77	68.1	0.5226
	Depression	184	53.0	67	59.8	0.2091
Baseline measures						
	Height (cm)	407	166.9 (9.4)	127	167.6 (9.4)	0.2308
	Weight (kg)	407	136.5 (29.2)	127	139.5 (26.5)	0.1312
	BMI (kg/m^2^)	407	48.5 (8.1)	127	49.4 (7.9)	0.2443
	EBW (kg)	407	66.7 (25.3)	127	69.0 (23.8)	0.2344
	HbA1c	195	6.1 (1.1)	64	6.1 (1.1)	0.6093
	Systolic blood pressure	407	126.7 (13.7)	123	128.7 (13.7)	0.1053
	Diastolic blood pressure	407	78.0 (8.3)	123	77.5 (7.8)	0.4733
	Beck Depression Inventory	303	10.4 (9.0)	105	9.4 (105)	0.5659
Year 1 measures						
	Weight (kg)	188	90.5 (20.6)	36	103.2 (23.2)	0.0006*
	BMI (kg/m^2^)	188	32.8 (5.9)	36	37.4 (7.6)	0.0004*
	EBW (kg)	188	21.4 (17.0)	36	33.7 (21.0)	0.0003*
	%EWL	188	71.8 (16.8)	36	51.1 (16.6)	< 0.0001*

Independent *t* test was used for assessing association between %EWL and variables with two groups (surgery type, gender, ethnicity, education level, state, marital status, diagnosed T2D, diagnosed hypertension, diagnosed depression). ANOVA was used testing for testing hypothesis of equality among more than two groups of categorical variables (education and marital status), and Spearman’s Rho was used for examining correlation of %EWL with continuous variables (age, % attended follow-up, systolic blood pressure, diastolic blood pressure, HbA1c, and BDI). Fisher’s exact test used for cell sizes < 5

* Significant at < 0.05 level

**Table 2:** Values between percent excess weight loss and other possible associated variables for entry into ANOVA and ANCOVA models

Success variable	Covariates	Test effect	*p* value
%EWL			
Categorical	Surgery type	− 6.900	< 0.0001**
	Gender	1.274	0.2070
	Ethnicity	0.397	0.6973
	Education level	0.455	0.7141
	State	− 0.106	0.9160
	Marital Status	1.966	0.1202
	Diagnosed T2D	− 4.015	< 0.0001**
	Diagnosed hypertension	− 2.235	0.0274*
	Diagnosed depression	− 2.913	0.0040**
Continuous	Age	− 0.258	< 0.0001**
	% attended follow-up	− 0.520	0.4375
	Systolic blood pressure	− 0.752	0.2625
	Diastolic blood pressure	− 0.012	0.8545
	HbA1c	− 0.313	0.0002**
	BDI	− 0.005	0.9469

Independent *t* test was used for assessing association between %EWL and variables with two groups (surgery type, gender, ethnicity, education level, state, marital status, T2D, diagnosed hypertension, diagnosed depression). ANOVA was used testing for testing hypothesis of equality among more than two groups of categorical variables (education and marital status), and Spearman’s Rho was used for examining correlation of %EWL with continuous variables (age, % attended follow-up, systolic blood pressure, diastolic blood pressure, HbA1c, and BDI).

* Significant at < 0.05 level

** Significant at < 0.01 level

**Table 3:** Models and Figs. 1–3: ANOVA model building and figures of surgery, T2D, and depression relationship with %EWL outcome

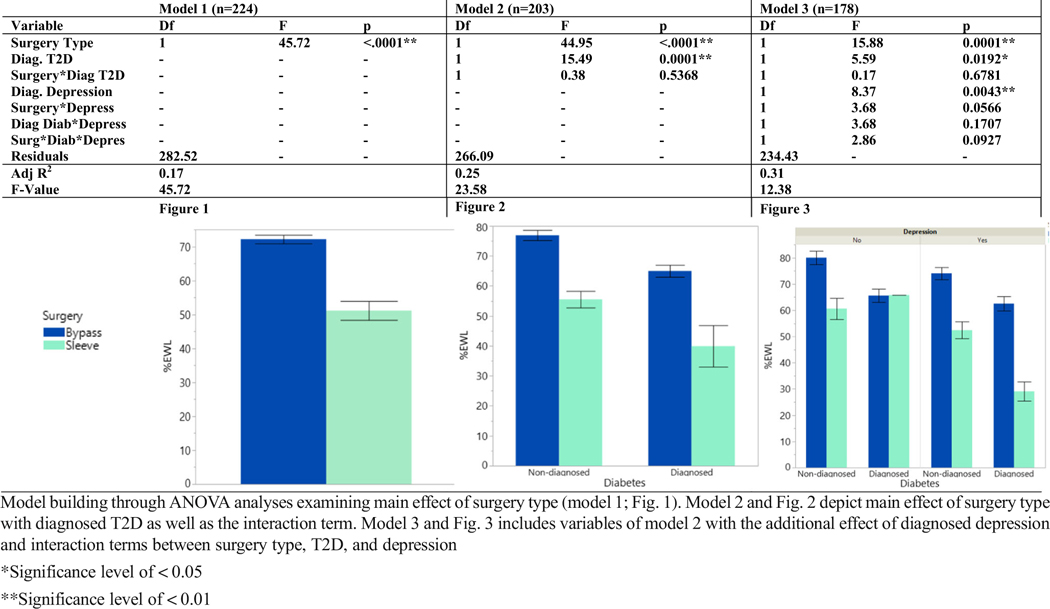

**Table 4:** Models and Figs. 4 and 5: ANCOVA model building and figures of surgery, HbA1c, and depression relationship with %EWL outcome